# Original W′‐balance models do not transfer to high‐intensity interval training in trained adult runners: Proposal of a single‐coefficient linear alternative

**DOI:** 10.14814/phy2.71152

**Published:** 2026-10-20

**Authors:** Felipe García‐Pinillos, Diego Jaén‐Carrillo, Leonardo Riquelme‐Sebastián, Santiago Alejo Ruiz‐Alias, Iván Fernández‐Navarrete, Fernando González‐Mohino, Violeta Muñoz de la Cruz, Javier Olaya‐Cuartero

**Affiliations:** ^1^ Department of Physical Education and Sports, Faculty of Sport Sciences Sport and Health University Research Institute (iMUDS), University of Granada Granada Spain; ^2^ Department of Physical Education, Sport and Recreation Universidad de La Frontera Temuco Chile; ^3^ Department of Sport Science University of Innsbruck Innsbruck Austria; ^4^ Department of Physical Education and Sports, Faculty of Education University of Cádiz Puerto Real Cádiz Spain; ^5^ Sport Training Laboratory, Faculty of Sport Sciences University of Castilla‐La Mancha Toledo Spain; ^6^ Research Group in Health, Physical Activity, and Sports Technology, Faculty of Education University of Alicante Sant Vicent del Raspeig Spain

**Keywords:** endurance running, high‐intensity interval training, modeling, power‐duration curve

## Abstract

This study evaluated the off‐the‐shelf predictive accuracy of four original W′‐balance recovery models (Morton and Billat linear, W′_BAL‐INT_, W′_BAL‐ODE_ and W′_BAL‐CAEN_) and proposed a single‐coefficient modified linear formulation (Linear__mod_) for predicting high‐intensity interval training (HIIT) performance with passive recovery in trained runners. Twenty‐seven trained runners completed a 9/3‐min running critical power (CP) test and two open‐ended HIIT sessions to volitional exhaustion at 108% CP with a 2:1 work: rest ratio. Work intervals were individualized so that energy expenditure above CP equalled 40% (HIIT40%) or 60% (HIIT60%) of W′. The original models were applied using their published parameters. All substantially over‐predicted the number of intervals completed (pooled MAE 26.4–72.4 intervals; bias +20.1 to +72.4). Linear__mod_ with its default recovery coefficient (*R* = 1) achieved a MAE of 4.85 intervals. Optimizing *R* at the population level (*R* = 1.63) reduced MAE to 2.85 intervals, while athlete‐specific optimisation (*R* = 1.54 ± 0.29) reduced MAE to 0.55 intervals. Individual *R* values were stable between protocols (within‐subject CV = 9.9%). Original W′‐balance models did not accurately predict running HIIT performance, whereas Linear__mod_ provided high predictive accuracy using a single athlete‐specific recovery coefficient.

## 1 INTRODUCTION

Power‐based training has long been established in cycling, where bike‐mounted power meters have enabled direct, second‐by‐second quantification of mechanical work and have transformed the way training intensity is prescribed and monitored (Allen & Coggan, 2006). In running, the development of foot‐mounted inertial measurement units capable of estimating mechanical power output in real time has opened similar opportunities only relatively recently. The Stryd foot pod is certainly the most extensively studied of these devices and the reliability and validity of running power data from that sensor have been documented across a range of running speeds, gradients and terrains (Navalta et al., 2019; Taboga et al., 2022), allowing power‐based intensity zones, training load metrics and performance models originally developed in cycling to be progressively translated to the running context (Cerezuela‐Espejo et al., 2020). Among these frameworks, the two‐parameter critical power (CP) model has emerged as particularly influential for characterizing endurance performance and individualizing training prescription (Jones et al., 2010). The model describes the relationship between sustainable power output and exercise duration through two physiologically interpretable parameters: CP, the asymptote of the power‐duration curve, representing the upper boundary of sustainable work and the highest power at which a metabolic steady state can be maintained; and W′, the finite amount of work that can be performed above CP before task failure (Jones et al., 2010; Poole et al., 2016). CP marks the boundary between the heavy and severe intensity domains, whereas W′ behaves as an energetic reservoir whose depletion is closely linked to the attainment of maximal oxygen uptake and to the development of intolerable fatigue (Jones et al., 2010). Both parameters can be derived from a series of constant‐power trials to exhaustion (Ruiz‐Alias et al., 2022) or, more recently, from simplified two‐point field protocols such as the 9/3‐min running CP test, whose validity and reliability have been established in trained runners (Ruiz‐Alias et al., 2022; Ruiz‐Alias et al., 2024a, 2024b).

While CP behaves as a comparatively stable index of cardiorespiratory fitness, with a standard error of the estimate (SEE) consistently below ≈3% (Ruiz‐Alias et al., 2024b), W′ has been shown to be a markedly less reproducible quantity (Ruiz‐Alias et al., 2024c). In an outdoor track‐running protocol involving repeated maximal time trials of 3, 5, 10 and 20 min duration, Ruiz‐Alias et al. (2024c) reported a SEE of 12.3% for W′ versus 2.4% for CP, with statistically detectable departures of the work actually performed above CP from the value predicted by the 2‐parameter model in the 10‐ and 20‐min trials. Accordingly, the authors argued that W′ behaves more as a variable than as a constant across different exhaustive trial durations. This greater variability has historically limited the direct use of W′ in training prescription. However, the formalization of so‐called W′‐balance models has transformed this picture: by tracking the dynamic depletion and reconstitution of W′ during intermittent exercise, these mathematical formulations allow coaches and scientists to anticipate the number of intervals an athlete should be able to complete at a given target power, total session duration and time to exhaustion (Skiba & Clarke, 2021). Such predictions enable high‐intensity interval training (HIIT) to be prescribed with sub‐interval accuracy, with individualized progression and objective monitoring of acute and chronic responses to training (Skiba et al., 2014), features that are particularly attractive given the well‐established efficacy of HIIT for improving athletic performance in endurance runners (García‐Pinillos et al., 2017).

Several mathematical formulations of W′ reconstitution have been proposed over the past two decades (Skiba & Clarke, 2021). The linear model by Morton and Veronique Billat (2004) is the simplest of them, assuming that W′ depletes and recovers at fixed linear rates governed exclusively by the instantaneous power deficit above or below CP, with no externally fitted parameter. To address the curvilinear (i.e., exponential) recovery of W′ observed experimentally after exhaustive exercise (Ferguson et al., 2010), Skiba and colleagues (Skiba et al., 2012) introduced the integral form of the W′‐balance model (W′_BAL‐INT_), in which the remaining W′ is expressed as the convolution of past expenditure above CP with a decaying exponential kernel governed by a single time constant τ__W′_. A subsequent reformulation as a differential equation (W′_BAL‐ODE_ (Skiba et al., 2015)) was proposed to address some of the theoretical limitations of the integral form, providing a parameter‐free formulation in which the apparent time constant of recovery emerges directly from the athlete's own CP and W′. More recently, empirical evidence has indicated that W′ reconstitution after exhaustion is better described by a biexponential than by a monoexponential function (W′_BAL‐CAEN_ (Caen et al., 2019; Caen et al., 2021)), with distinct fast and slow recovery components hypothesized to reflect different physiological pools (e.g., phosphocreatine resynthesis and recovery of fast‐twitch fibers for the fast component; oxidative metabolism and recovery of slow‐twitch fibers for the slow component). Together, these four formulations represent the main mathematical approaches currently available for modeling W′ reconstitution during intermittent exercise (Skiba & Clarke, 2021).

Despite their conceptual maturity and practical appeal, the development and empirical validation of W′‐balance models have been almost exclusively confined to cycling, typically using ergometer‐based protocols with active recovery between work bouts (Caen et al., 2021; Morton & Veronique Billat, 2004; Skiba et al., 2012; Skiba & Clarke, 2021). The few attempts to apply these formulations to running have produced disappointing results: most notably, Galbraith et al. (2015) reported that the W′_BAL‐INT_ model, when applied to intermittent treadmill running using the cycling‐derived τ__W′_ relationship of Skiba et al. (2012), was unable to predict the runners' performance, and acceptable accuracy was only obtained when τ__W′_ was re‐fitted directly to the running data. Beyond this isolated study, no comprehensive evaluation of the off‐the‐shelf predictive accuracy of the available W′‐balance recovery models has been conducted in a HIIT protocol performed by trained runners with passive recovery, a common recovery modality during structured interval sessions in endurance running (Buchheit & Laursen, 2013). Moreover, no simple, athlete‐specific formulation has yet been proposed and validated for this experimental context, despite its evident practical relevance for the prescription of HIIT sessions in this population. Building on this gap, the present study aimed (i) to evaluate the off‐the‐shelf predictive accuracy of the four original W′‐balance recovery models (i.e., linear, monoexponential and biexponential approaches) in predicting the number of completed intervals during HIIT sessions with passive recovery in trained runners; and (ii) to propose a simple modification of the linear model (Linear__mod_), incorporating a single dimensionless recovery coefficient *R* fitted at both the population and individual levels, and to test its within‐subject stability across two HIIT protocols differing in per‐interval W′ depletion (40% and 60%).

## 2 METHODS

### 2.1 Study design

A cross‐sectional study with a within‐subject repeated‐measures design was conducted (Figure 1). Each participant attended three separate sessions, 3–5 days apart: (i) a baseline session for the determination of CP and W′, and (ii and iii) two open‐ended HIIT sessions performed to volitional exhaustion at 108% CP with two different per‐interval W′ depletion targets (HIIT40% and HIIT60%). The order of the experimental conditions was counterbalanced. All sessions were performed on a flat outdoor 325 m athletics track at similar ambient conditions and time of day, following a standardized 15‐min warm‐up. Participants avoided strenuous exercise in the 24 h prior to each session and replicated their pre session nutrition and caffeine intake.

**FIGURE 1:**
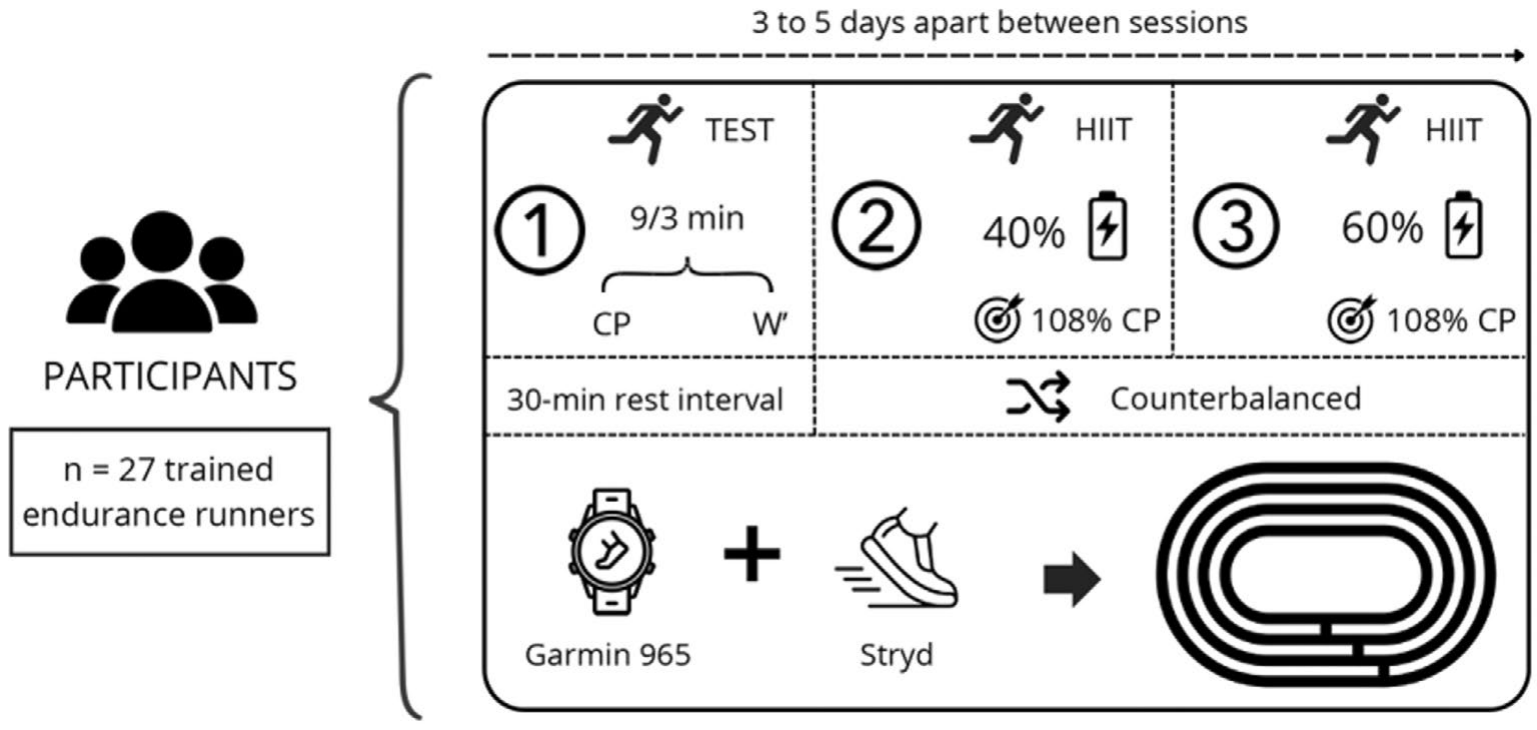
Illustration of the experimental procedure.

### 2.2 Participants

Twenty‐seven trained endurance runners (25 males, 2 females; age range 19–39 years) completed all study procedures and were retained for analysis. Participants were recruited via convenience sampling from local running clubs and training groups. Group means (± SD) were: body mass 68.7 ± 8.5 kg, height 177.0 ± 7.6 cm, running experience 8.0 ± 5.7 years and self‐reported training volume 35.5 ± 12.8 km·week^−1^. Inclusion criteria were: (i) ≥ 2 years of structured running training, and (ii) weekly training volume of ≥20 km·week^−1^. Exclusion criteria comprised any musculoskeletal injury or cardiovascular condition that could compromise maximal effort in the previous 6 months. All participants provided written informed consent. The study was approved by the Ethics Committee of the University of Granada (No. 243/2026) and conducted in accordance with the Declaration of Helsinki (World Medical Association, 2024).

### 2.3 Materials and test

Running power output was measured with a foot‐mounted inertial measurement unit (Stryd NextGen, Inc., Boulder, CO, USA). The reliability of the power data from this sensor has been validated in running conditions, with coefficient of variations <3.5% (Navalta et al., 2019).

Body mass and height of participants were measured using a weight and a height scale, respectively (Seca 813 and 213; Seca Ltd., Hamburg, Germany), during the initial testing session and maintained for subsequent sessions within the Stryd sensor. The Stryd was paired to a Garmin Forerunner 965 smartwatch (Garmin Ltd., Olathe, Kansas, USA).

The testing session started with a standardized warm‐up protocol consisting of 10 min of running at low intensity, then a series of dynamic mobility exercises and finally, three progressive runs of 100 m with 2 min of rest. Then, athletes performed a running test consisting of a 9/3 min CP test (9/3CP) that has been widely used in similar studies to determine CP and W′ in endurance runners (Ruiz‐Alias et al., 2022; Ruiz‐Alias et al., 2024a, 2024b). Participants performed a single testing session composed of two time trials of 9 and 3 min interspaced by a 30‐min rest interval. Subjects were asked to complete the longest distance possible in each time trial and the perceived effort, following each time trial, was evaluated using the Borg CR‐10 scale (Borg, 1970). The power outputs from both the 9‐ and 3‐min trials were recorded, together with running speed and distance covered. The sensor was attached to the laces of the right footwear during the testing protocol.

The 9/3CP test has been recently established as a valid two‐point configuration to describe the power‐duration relationship in running (Ruiz‐Alias et al., 2022; Ruiz‐Alias et al., 2025) and the two parameters, CP and W′, were subsequently determined using the inverse of time linear CP model (Ruiz‐Alias et al., 2023; Whipp et al., 1982). Therefore, the configuration of 9‐ and 3‐min time trials was chosen based on the established validity of CP (Ruiz‐Alias et al., 2022), the alignment of CP and W′ with those determined in a 5 time trial configuration through the same procedure (Ruiz‐Alias et al., 2023; Ruiz‐Alias et al., 2025), and the reliability of these parameters when considering different protocol configurations (i.e. trials order, day, or within session recovery) (Ruiz‐Alias et al., 2024a, 2024b).

### 2.4 Experimental procedures – high‐intensity interval sessions

Both HIIT sessions were performed at a target intensity of 108% CP (Ruiz‐Alias et al., 2022), which is the intensity associated with the maximal oxygen uptake (VO_2max_) and a typical intensity for HIIT workouts in endurance runners (García‐Pinillos et al., 2017). Target power was displayed as instantaneous, non‐averaged power on the paired Garmin watch. Participants, all experienced runners familiar with pace/power self‐regulation, were instructed to sustain 108% CP throughout each work interval on a flat outdoor track. Those experimental sessions consisted of an open‐ended series of work–recovery cycles. Work‐interval duration was individualized so that the per‐interval energy expenditure above CP equalled either 40% or 60% of the participant's W′ (HIIT40% and HIIT60%, respectively).

(1) $$W^{\prime }\;\text{depletion}=\left(P_{\_\text{work}}-\mathrm{CP}\right)\times t_{\_\text{work}}$$

where *P*__work_ is the target power output for work intervals (W), and *t*__work_ is the duration of each work interval (s). Rearranging Equation (1), the work‐interval duration prescribed for each athlete and protocol was:

(2) $$t_{\_\text{work}}=\left(f\cdot W^{\prime }\right)/\left(P_{\_\text{work}}-\mathrm{CP}\right)$$

where *f* is the target depletion fraction (0.40 for HIIT40% and 0.60 for HIIT60%), W*′* and CP are determined from the 9/3CP test, and *P*_*_*work_ the target power at 108% CP (W). Recovery duration was set to half of the work duration (work: rest ratio 2:1):

(3) $$t_{\_\text{rest}}=0.5\cdot t_{\_\text{work}}$$

where *t*_*_*rest_ is the duration of each (passive) recovery interval (s). Recovery was fully passive, ensuring that recovery power output was negligible relative to CP (*P*__rest_ *≈* 0 W).

Participants were instructed to complete as many intervals as possible while sustaining the target power. Each work interval was always completed in full before the participant decided whether to continue. The number of intervals completed (*N*) was the primary outcome. Note that this volitional stopping criterion differs conceptually from the energy‐based exhaustion criterion used in the simulations, in which exhaustion is defined by the instant at which modeled W′ reaches zero rather than by the participant's own judgment of their capacity to continue. All the sessions were conducted under similarly stable weather conditions, at the same time of day (±1 h), and with participants wearing the same racing footwear for all experimental sessions.

### 2.5 W′ recovery models

In the current study, the four main mathematical approaches that have been proposed to model W′ reconstitution during intermittent exercise were considered (Skiba & Clarke, 2021) and benchmarked against a novel linear formulation introduced in the present study (fifth approach).

- Linear model by Morton and Veronique Billat (2004). The simplest and oldest formulation, in which both depletion and reconstitution proceed at fixed linear rates determined exclusively by the power output and CP. The recovery rate during rest is set equal to the power deficit below CP (CP − *P*__rest_), so for passive recovery the model assumes that W′ is restored at a rate equal to CP itself. The model contains no free recovery parameter so, once CP, W′ and the protocol durations are specified, the predicted number of intervals is fully determined. The complete mathematical formulation is provided in Morton and Veronique Billat (2004).
- Monoexponential W′‐balance model in its integral form (W′_BAL‐INT_) (Skiba et al., 2012). The first of the two original W′ balance formulations, conceived as a refinement of the Morton–Billat linear model (Morton & Veronique Billat, 2004) to account for the curvilinear (i.e., exponential) recovery of W′ observed experimentally after exhaustive exercise (Skiba & Clarke, 2021). The model expresses the remaining W′ as the convolution of the past expenditure above CP with a decaying exponential kernel, governed by a time constant τ__W′_ that depends on the average power deficit below CP during recovery. A defining feature of this formulation is the assumption of “ongoing recovery” which means that every previously expended joule of W′ is being reconstituted at every subsequent time point, even while additional W′ is still being expended above CP (Skiba & Clarke, 2021). The complete mathematical formulation is provided in Skiba et al. (2012).
- Monoexponential W′‐balance model in its differential form (W′_BAL‐ODE_) (Skiba et al., 2015). Developed subsequently to address some of the theoretical limitations of W′_BAL‐INT_ (Skiba & Clarke, 2021), this formulation specifies W′ kinetics as a piecewise first‐order ordinary differential equation in which depletion and recovery are mutually exclusive: W′ is only consumed when P > CP and only reconstituted when P < CP. A second distinguishing feature is that the apparent time constant of recovery emerges directly from the athlete's CP and W′, without any externally fitted parameter. The complete mathematical formulation is provided in Skiba et al. (2015).
- Biexponential W′‐balance Caen model (W′_BAL‐CAEN_) (Caen et al., 2019; Caen et al., 2021). A two‐component extension of the exponential framework, motivated by empirical evidence that W′ reconstitution after exhaustive exercise follows a sum of two exponentials rather than a single exponential (Caen et al., 2019; Caen et al., 2021). The model decomposes recovery into a fast and a slow component, hypothesized to reflect distinct physiological pools (e.g., phosphocreatine resynthesis and fast‐twitch fiber recovery for the fast component, and oxidative metabolism and slow‐twitch fiber recovery for the slow component). The reference values used here for the amplitudes and time constants of the two components were those reported by Caen et al. (2019, 2021) for passive recovery in trained cyclists (A__F_ = 0.30, τ__F_ = 22 s; A__S_ = 0.70, τ__S_ = 377 s). The complete mathematical formulation is provided in Caen and colleagues (2019 and 2021) (Caen et al., 2019; Caen et al., 2021).

Of note, some common implementations. First, all four models share a common depletion assumption: during work intervals above CP, W′ is consumed at a rate equal to the power deficit above CP (P − CP) and differ exclusively in the way W′ is reconstituted during the subsequent recovery phase. Second, consistent with the prescribed protocol, each work interval was modeled as a constant power output equal to 108% CP and each rest interval as passive (*P*__rest_ ≈ 0 W). Each model was applied iteratively across the successive work–recovery cycles of every session, using each athlete's individually determined CP and W′ from the 9/3CP test, and run until the predicted W′ first reached zero during a work interval (the exhaustion criterion that defined the simulated number of completed intervals, *N*). To prevent runaway predictions when modeled recovery exceeded depletion (a behavior formally possible for several of the models under passive recovery), the simulation was capped at 80 intervals.

v A novel linear model with tunable recovery coefficient (Linear__mod_). As a fifth approach, we propose a one‐parameter generalization of the Morton–Billat linear model (Morton & Veronique Billat, 2004) in which the recovery rate during rest is re‐parameterised as a fitted multiple of the work‐rate deficit (*P*__work_ − CP), rather than being fixed by CP alone. The model retains the linear‐bucket simplicity of the original Morton–Billat formulation but introduces a single tunable parameter (i.e., a dimensionless recovery coefficient *R*) that is estimated from observed performance data. This modification was motivated by the fact that the original linear model becomes degenerate for the present HIIT protocols, since the passive‐rest durations (76 ± 37 s in HIIT40%; and 116 ± 55 s and HIIT60%) exceed W′/CP (≈32 s on average) and therefore predict complete W′ replenishment between intervals and an unbounded number of repetitions. Anchoring the recovery rate to the work‐rate deficit rather than to CP itself avoids this degeneracy and allows the model to be calibrated to the recovery dynamics actually observed in the data. The procedure used to estimate *R* at the population and individual levels is described in the Statistical analysis section.

### 2.6 Statistical analysis

Descriptive statistics are reported as mean ± standard deviation (SD). Predictive accuracy of each W′ balance model was quantified by comparing the predicted and observed number of completed intervals through the mean absolute error (MAE) and the mean error (ME, used as the bias indicator) (Atkinson & Nevill, 1998; Hopkins, 2000). Both metrics were computed pooled across the 54 sessions and stratified by HIIT protocol (HIIT40% and HIIT60%). Positive ME values denote over‐prediction and negative values denote under‐prediction. Level 1, off‐the‐shelf evaluation. The four original models (i.e., Morton & Billat (Morton & Veronique Billat, 2004), W′_BAL‐INT_ (Skiba et al., 2012), W′_BAL‐ODE_ (Skiba et al., 2015) and W′_BAL‐CAEN_ (Caen et al., 2021)) were applied using their original published parameter values, with no tuning to the present data. Levels 2 and 3, population‐ and individual‐optimal *R* for the Linear__mod_ model. The recovery coefficient *R* was estimated by minimizing MAE via a fine‐grid search across the physiologically plausible range (0.10–4.10) in steps of 0.005. At level 2, a single *R* was fitted to the pooled set of 54 sessions. At level 3, an athlete‐specific *R* was fitted by minimizing the sum of absolute errors across each athlete's two sessions. The fitted *R* values were then applied to recompute predicted *N*, and the corresponding MAE and ME were derived as described above. Finally, for the 27 athletes who completed both protocols, the stability of the individually‐fitted *R* between HIIT40% and HIIT60% was assessed by the within‐subject coefficient of variation (CV%) (Atkinson & Nevill, 1998; Hopkins, 2000). All analyses were conducted using R software (R Foundation for Statistical Computing, Vienna, Austria).

## 3 RESULTS

Table 1 shows the results obtained during the 9/3CP test. The 9‐ and 3‐min running time trials were performed at near‐maximal intensities, with post‐effort RPE scores of 9.4 ± 0.6 and 9.6 ± 0.6 on the Borg CR‐10 scale, respectively. Mean running power was 344 ± 57 W (5.02 ± 0.54 W·kg^−1^) over the 9‐min effort and 380 ± 58 W (5.54 ± 0.59 W·kg^−1^) over the 3‐min effort. From the linear power vs. 1/time model fitted to these two trials, CP averaged 327 ± 58 W (4.76 ± 0.55 W·kg^−1^) and the W′ averaged 9550 ± 3976 J (142 ± 65 J·kg^−1^).

**TABLE 1:** Performance (mean ± SD) during the 9‐ and 3‐min maximal running test and derived critical power (CP) parameters.

Variable	9‐min effort	3‐min effort
Distance covered (m)	2487 ± 216	923 ± 87
Mean speed (km·h^−1^)	16.58 ± 1.44	18.47 ± 1.73
Mean pace (min:s·km^−1^)	3:37	3:15
Mean power, absolute (W)	344 ± 57	380 ± 58
Mean power, relative (W·kg^−1^)	5.02 ± 0.54	5.54 ± 0.59
Post‐effort RPE (CR‐10)	9.4 ± 0.6	9.6 ± 0.6
*Derived from the power versus 1/time model*		
CP (W)	327 ± 58	
CP (W·kg^−1^)	4.76 ± 0.55	
Work capacity above CP, W′ (J)	9550 ± 3976	
Work capacity above CP, W′ (J·kg^−1^)	142 ± 65	

Table 2 summarizes the main characteristics of the experimental running sessions performed (HIIT40% and HIIT60%). A total of 54 HIIT sessions (27 at the 40% and 27 at the 60% protocol) were available for analysis. The protocols differed in the severity of each work bout: in the 40% condition each work interval depleted on average 40.0% ± 0.6% of W′ (corresponding to 152 ± 75 s of running at 108% CP), whereas in the 60% condition it depleted 60.0% ± 1.1% of W′ (233 ± 110 s of work). Participants completed 9.6 ± 4.7 intervals in the HIIT40% and 5.5 ± 3.5 in the HIIT60%.

**TABLE 2:** Characteristics of the two HIIT protocols (40% and 60% W′ depletion).

Variable	HIIT 40%	HIIT 60%
Work interval duration (s)	152 ± 75	233 ± 110
Rest interval duration (s)	76 ± 37	116 ± 55
Work:rest ratio	2:1	2:1
Target running power (W)	353 ± 62	351 ± 62
Target intensity (%CP)	108	108
W′ depleted per work interval (J)	3816 ± 1591	5832 ± 2336
W′ depleted per work interval (%W′)	40.0 ± 0.6	60.0 ± 1.1
Intervals completed (*n*)	9.6 ± 4.7	5.5 ± 3.5
Total work time per session (min)	22.6 ± 13.3	19.1 ± 9.1
Total session duration (min)	32.6 ± 19.6	26.7 ± 13.2

Table 3 shows the estimation of completed intervals from four different W‐balance recovery models, in its original form. Each model was applied to the 54 HIIT‐running sessions using the formulations and reference parameter values as published.

**TABLE 3:** Predicted versus observed number of completed intervals (*N*) using four original W′‐balance recovery model formulations applied with their published parameters.

Model	Parameter(s) used	HIIT40% (*N*, intervals)	HIIT60% (*N*, intervals)	All sessions (*N*, intervals)
Observed	—	9.6 ± 4.7	5.5 ± 3.5	7.6 ± 4.6
Linear (Morton & Veronique Billat, 2004)	None (deterministic; recovery rate = CP·*t*__rest_)	Capped at *N* = 80^a^		
W′_BAL‐INT_ (Skiba et al., 2012)	τ__W_′ = 546·e^a^(−0.01·CP) + 316 (mean ≈ 337 s)	68.7 ± 27.6	68.2 ± 28.3	68.5 ± 27.7
W′_BAL‐ODE_ (Skiba et al., 2015)	None (τ__app_ = W′/CP; mean ≈ 31 s)	Capped at *N* = 80^a^		
W′_BAL‐CAEN_ (Caen et al., 2021)	A__F_ = 0.30, τ__F_ = 22 s; A__S_ = 0.70, τ__S_ = 377 s	43.8 ± 38.3	11.0 ± 25.4	27.7 ± 36.3

Abbreviations: A_F/τ_F and A_S/τ_S, amplitudes and time constants of the fast and slow components of the bi‐exponential recovery curve; CP, critical power; DCP, average power deficit below CP during recovery (= CP for passive recovery); W′, finite work capacity above CP.

^a^ The simulation was capped at *N* = 80 to avoid runaway predictions.

Table 4 shows the errors reported by the different models analyzed. The Morton and Billat linear model predicted complete W′ replenishment within every rest interval. All rest intervals, 26–260 s, exceeded W′/CP, which averaged ≈32 s in the present cohort, and consequently returned the simulation cap (*N* = 80) in 54/54 sessions, with a mean over‐prediction of +72.4 intervals (MAE 72.4). The W′_BAL‐ODE_ model, whose apparent time constant emerges from W′/CP, produced an identical pattern: 54/54 sessions at the simulation cap, MAE 72.4, bias +72.4 intervals. The W′_BAL‐INT_ model, applied in its original convolution form with τ__W′_ derived from CP for passive recovery (DCP = CP; mean τ__W′_ ≈ 337 s), saturated at the simulation cap in 46/54 sessions with a mean over‐prediction of +60.9 intervals (MAE 63.1). The W′_BAL‐CAEN_ model produced the smallest off‐the‐shelf error of the four formulations (MAE 26.4 intervals; bias +20.1) but still substantially over‐predicted *N* and it saturated at the simulation cap in 17/54 sessions. Errors were highly protocol‐dependent: in the HIIT40%, the model over‐predicted *N* by +34.2 intervals on average (MAE 40.3); in the HIIT60%, bias dropped to +5.5 intervals and MAE to 12.0.

**TABLE 4:** Absolute and mean errors reported for the different W′‐balance recovery model formulations.

Model	HIIT40% MAE/bias	HIIT60% MAE/bias	All sessions MAE/bias
Linear (Morton & Veronique Billat, 2004)	70.4/+70.4	74.5/+74.5	72.4/+72.4
W′_BAL‐INT_ (Skiba et al., 2012)	61.4/+59.1	64.8/+62.7	63.1/+60.9
W′_BAL‐ODE_ (Skiba et al., 2015)	70.4/+70.4	74.5/+74.5	72.4/+72.4
W′_BAL‐CAEN_ (Caen et al., 2019; Caen et al., 2021)	40.3/+34.2	12.0/+5.5	26.4/+20.1

*Note*: Errors are expressed in number of intervals. Bias = mean error = predicted − observed (positive values indicate over‐prediction).

Abbreviation: MAE, mean absolute error.

Table 5 shows the analysis of the errors reported by the Linear__mod_ model. In this case, the *R* coefficient was obtained as a population‐optimal parameter from our own group of athletes. It is a single value of the recovery parameter that minimized the MAE when applied uniformly to all 54 sessions. The optimal linear coefficient was *R* = 1.63.

**TABLE 5:** Off‐the‐shelf (*R* = 1) and population‐optimal recovery parameters (Level 2) for the modified Linear model (Linear__mod_).

Model	Population‐optimal parameter	MAE (intervals)	Bias (ME, intervals)
Linear__mod_	*R* = 1 (off‐the‐shelf)	4.85	—
Linear__mod_	*R* = 1.63	2.85	−1.19

Abbreviations: MAE, mean absolute error; ME, mean error (bias = predicted − observed; negative values indicate the model under‐predicted the number of completed intervals, positive values indicate over‐prediction); *R*, dimensionless recovery coefficient of the linear W′‐balance model.

Table 6 summarizes the stability of the individually‐fitted recovery parameter across the two HIIT protocols in the 27 athletes who completed both conditions. The Linear__mod_ coefficient *R* was stable. Similar values were obtained in the 40% and 60% protocols (1.53 vs. 1.58), with a small paired difference (~0.05) and a CV of 9.9%.

**TABLE 6:** Stability of the individually‐fitted parameter (*R*) across the 40% and 60% depletion conditions in paired participants (mean ± SD).

	HIIT40%	HIIT60%	Mean of both protocols	Within‐subject CV (%)
Linear__mod_	1.53 ± 0.31	1.58 ± 0.27	1.56 ± 0.25	9.9 ± 16.3

Abbreviations: CV (%), coefficient of variation expressed in percentage; HIIT40%, experimental protocol based on a 40% W′ depletion; HIIT60%, experimental protocol based on a 60% W′ depletion.

Table 7 shows the individual‐optimal recovery parameters and its associated errors for the Linear__mod_ model. The Linear__mod_ model reached a pooled MAE of 0.55 intervals (bias = −0.36). Individual‐optimal *R* values ranged from 0.63 to 1.89 (mean 1.54 ± 0.29), with the cohort median (1.63) almost identical to the single population‐optimal value (*R* = 1.63; Table 5). The discrepancy between protocols was small, with larger errors in the HIIT60% than in the HIIT40% (MAE 0.00 vs. 1.12 intervals, respectively).

**TABLE 7:** Predictive accuracy of the modified Linear model (Linear__mod_) based on a individual‐optimal *R* coefficient.

Condition	Individual‐optimal *R* ^a^	MAE (intervals)	Bias (ME, intervals)
Pooled (40% + 60%)	1.54 ± 0.29	0.55	−0.36
HIIT40%		0.00	+0.00
HIIT60%		1.12	−0.73

Abbreviations: CP, critical power; MAE, mean absolute error; ME, mean error (predicted − observed); W′, finite work capacity above CP.

^a^ *R* was fitted jointly across each athlete's two sessions (HIIT40% and HIIT60% combined), not separately per protocol.

## 4 DISCUSSION

The aim of the present study was twofold: to evaluate the off‐the‐shelf predictive accuracy of the four original W′‐balance recovery models in a HIIT context in trained runners with passive recovery, and to propose a simple modified linear formulation (Linear__mod_) tailored to this context. The main finding is that none of the four original formulations (Caen et al., 2019; Caen et al., 2021; Morton & Veronique Billat, 2004; Skiba et al., 2012; Skiba et al., 2015), when applied with their published parameters, was able to reproduce the observed number of completed intervals in a practically meaningful way: pooled mean absolute errors ranged from 26 to 72 intervals, while observed *N* averaged 7.6 ± 4.6 across the 54 sessions analyzed. In contrast, the proposed Linear__mod_, incorporating a single dimensionless recovery coefficient *R* fitted at either the population or the individual level, reduced this error to 2.85 and 0.55 intervals, respectively, and the individually fitted *R* remained stable between the two HIIT protocols tested (mean within‐subject CV ≈ 10%), supporting its use as an athlete‐specific parameter for HIIT prescription.

The systematic over‐prediction of *N* by all four original models reflects, in each case, a specific mismatch between the model's assumptions and the conditions of the present protocols. The Morton–Billat linear model (Morton & Veronique Billat, 2004) and the W′_BAL‐ODE_ (Skiba et al., 2015) share a common structural problem when applied to passive recovery: both predict a recovery rate that is too fast to be consistent with the observed time‐to‐exhaustion. In the Morton‐Billat formulation, the recovery rate during passive rest equals CP itself, so that any rest interval longer than W′/CP seconds, approximately 32 s on average in the present cohort, replenishes W′ completely, producing an unbounded number of repetitions regardless of work‐bout severity. In the W′_BAL‐ODE_, the apparent time constant of recovery (τ__app_ = W′/CP) averaged only 31 s, so that the 26–260 s rest intervals of the present protocols (i.e., HIIT40%: 26–175 s whereas HIIT60%: 40–260 s) allowed recovery of more than 91% of the deficit per cycle, again precluding W′ exhaustion within any plausible number of intervals. The W′_BAL‐INT_ (Skiba et al., 2012), although based on a markedly slower time constant (τ__W′_ ≈ 337 s for the cohort mean), saturated at the simulation cap in 46 of 54 sessions due to its central assumption of “ongoing recovery during exercise”: the convolution‐based formulation continually attenuates W′ depletion during work bouts, allowing a steady‐state W′ balance well above zero to be maintained across successive work–rest cycles. This is the same theoretical concern recently raised by Skiba and Clarke (2021) themselves, who noted that the integral formulation predicts time‐to‐exhaustion values longer than those allowed by the underlying two‐parameter CP model under continuous high‐intensity exercise. Finally, the W′_BAL‐CAEN_ model (Caen et al., 2019; Caen et al., 2021), with its fast component (A__F_ = 0.30, τ__F_ = 22 s), reconstitutes approximately 96% of the fast pool within 80 s of rest, producing recovery dynamics that are again incompatible with the observed exhaustion patterns. It is worth noting that the parameters proposed by Caen et al. (2021) were derived from cyclists during isolated recovery curves following a single exhaustive effort and not for iterative prediction in a HIIT context, so their direct transfer to running with passive recovery between brief work bouts was a priori questionable.

Our findings are consistent with, and extend, the only previous attempt to apply a W′‐balance formulation to intermittent running. Galbraith et al. (2015) reported that the W′_BAL‐INT_, when implemented with the cycling‐derived τ__W′_ relationship of Skiba et al. (2012), was unable to predict intermittent treadmill running performance, and acceptable accuracy was only obtained when τ__W′_ was re‐estimated from the runners' own data. Our results corroborate and substantially broaden that observation in three respects. First, they demonstrate that the off‐the‐shelf failure is not specific to the W′_BAL‐INT_ but is a common feature of all four W′‐balance formulations evaluated in the present study. Second, they show that the failure occurs not only in treadmill running with active recovery, the condition tested by Galbraith and colleagues, but also, and even more markedly, in outdoor track running with passive recovery, which is a very common recovery modality in structured HIIT sessions for endurance runners (Buchheit & Laursen, 2013; García‐Pinillos et al., 2017). Third, they show that the magnitude of the off‐the‐shelf prediction error in running HIIT is substantial, exceeding an order of magnitude for three of the four formulations. Together, these observations support the case for proposing a new formulation tailored to running HIIT sessions as an alternative to the existing ones.

The proposed Linear__mod_ was designed to retain the parsimony and conceptual simplicity of the original Morton‐Billat linear model (Morton & Veronique Billat, 2004) while addressing its degeneracy under passive recovery. By re‐parameterising the recovery rate as a fitted multiple of the work‐rate deficit, *R* · (*P*__work_ − CP), rather than as the fixed (CP − *P*__rest_) of the original formulation, the recovery rate is decoupled from the absolute magnitude of CP and constrained to physiologically plausible values when calibrated to data. Already with its off‐the‐shelf parameterisation (*R* = 1, which under the 2:1 work:rest ratio used here restores only half of the W′ expended during the preceding work interval), Linear__mod_ outperformed all four original models by an order of magnitude (MAE 4.85 vs. 26–72 intervals). With a single population‐optimal coefficient (*R* = 1.63) fitted to the entire cohort, the error fell to 2.85 intervals and the systematic bias was largely eliminated, indicating that trained runners require a recovery rate that is approximately 1.6 times the work‐rate deficit per second of passive rest, which is substantially slower than implied by either the Morton‐Billat or the W′_BAL‐ODE_ formulations and broadly consistent in magnitude with the curvilinear recovery dynamics reported in cycling for similar work intensities (Ferguson et al., 2010). The further reduction of MAE to 0.55 intervals with athlete‐specific *R* confirms that the residual error of the population‐level model is dominated by between‐subject variability in the recovery rate, an observation that is in turn consistent both with the marked inter‐individual differences in W′ reconstitution kinetics previously reported in cyclists (Skiba et al., 2014) and with the substantial intra‐individual variability of W′ itself across protocols and conditions (Ruiz‐Alias et al., 2024c).

A defining feature of the proposed Linear__mod_ is that the individually fitted *R* proved markedly stable between the two protocols tested, with a within‐subject coefficient of variation below 10%. This is non‐trivial because the 40% and 60% protocols differed substantially in both work‐bout duration (≈152 s vs. ≈233 s) and rest duration (≈76 s vs. ≈116 s), so a protocol‐dependent artifact would have produced a much larger discrepancy. The observed stability indicates that *R* behaves as a reproducible, athlete‐specific calibration coefficient rather than a protocol‐dependent artifact. However, given the algebraic structure of the present experimental design, *R* should be regarded as an empirical tolerance coefficient of the model rather than as direct evidence of an underlying physiological trait. Whether it reflects specific determinants of W′ reconstitution such as muscle‐fiber composition, capillary density, mitochondrial oxidative capacity, or VO_2_ kinetics (Ferguson et al., 2010; Skiba et al., 2015) remains speculative and was not directly tested in the present study. Taken together, these observations support the use of a single, athlete‐specific *R*, fitted from a small number of HIIT sessions, as the simplest formulation that suffices to predict running HIIT performance under the experimental conditions investigated.

Finally, some limitations must be addressed. The proposed Linear__mod_ model was developed and calibrated exclusively for HIIT sessions performed at 108% CP with passive recovery and a 2:1 work:rest ratio in trained endurance runners. Three caveats limit the generalisability of the present findings. First, the model assumes that *R* is independent of *P*__work_. While our cross‐protocol analysis supports *R*‐stability between two W′ depletion targets at the same target intensity, the validity of a single athlete‐specific *R* across markedly different work intensities (e.g., 105% CP vs. 120% CP) has not been tested and cannot be assumed. Second, the Linear__mod_ recovery equation does not include the recovery power output (*P*__rest_) as an explicit term, and therefore cannot in its present form discriminate passive from active recovery. Extending the model to active recovery (e.g., dW′/dt = *R* · (*P*__work_ − *P*__rest_)) is a plausible avenue but would require empirical validation. Third, the value of *R* = 1.63 derived for the present cohort of trained runners should not be transferred to other populations (recreational runners, elite athletes, or other modes of exercise) without dedicated calibration. The methodological framework (i.e., a one‐parameter linear recovery model with athlete‐specific *R* fitted from a small number of HIIT sessions) is, however, in principle transferable across populations and protocols. Fourth, the reported errors for the population‐ and athlete‐specific *R* (2.85 and 0.55 intervals, respectively) were obtained by estimating and evaluating *R* on the same sessions, and therefore represent in‐sample calibration error rather than out‐of‐sample predictive accuracy. Future studies could addressed a dedicated out‐of‐sample validation using a third, independent HIIT sesión.

## 5 CONCLUSION

The four original W′‐balance recovery models, when applied off‐the‐shelf with their published parameters, substantially mispredict the number of completed intervals during HIIT performed at 108% CP with passive recovery in trained runners. These findings demonstrate that cycling derived W′‐balance formulations do not generalize to the present running context. The proposed Linear__mod_ substantially improved predictive accuracy across all parameterisation levels. With a single population‐optimal coefficient (*R* = 1.63), Linear__mod_ reduced the mean absolute fitting error to 2.85 intervals, whereas based on an athlete‐specific calibration, this error fell below one interval (MAE = 0.55). The individually fitted *R* remained stable within athletes across the two HIIT protocols tested (mean within‐subject CV <10%), supporting its interpretation as an athlete‐specific parameter rather than a protocol‐dependent artifact. Taken together, the results indicate that a simple, single‐parameter linear formulation, when calibrated to the individual runner, provides useful predictions of HIIT tolerance under the experimental conditions investigated.

## AUTHOR CONTRIBUTIONS

**Felipe García‐Pinillos:** Conceptualization; data curation; formal analysis; investigation; methodology. **Diego Jaén‐Carrillo:** Conceptualization; data curation; formal analysis; methodology. **Leonardo Riquelme‐Sebastián:** Formal analysis; investigation. **Santiago Alejo Ruiz‐Alias:** Conceptualization; formal analysis; visualization. **Iván Fernández‐Navarrete:** Formal analysis; investigation; resources; validation. **Fernando González‐Mohino:** Data curation; formal analysis; investigation; visualization. **Violeta Muñoz de la Cruz:** Investigation; methodology; visualization. **Javier Olaya‐Cuartero:** Formal analysis; investigation; methodology.

## FUNDING INFORMATION

This research received no specific grant from any funding agency in the public, commercial, or not‐for‐profit sectors.

## CONFLICT OF INTEREST STATEMENT

There are no conflicts to report.

## CODE AVAILABILITY STATEMENT

Any custom computer code or algorithm that is not publicly available and has been used to generate results that are reported in the paper and are central to its main claims, must be made available for peer‐review (e.g., through a private link at an appropriate repository as described below). Any reason that would preclude the need for code or algorithm sharing will be evaluated by the editors. For any study where use of a custom code or a mathematical algorithm is central to the results and conclusions, for publication the code should be made available in a public DOI‐minting repository such as Zenodo or Code Ocean or GitHub and cited in the reference list. Details about how the code can be accessed and any restrictions should be stated in a Code Availability statement.

## Data Availability

The data that support the findings of this study are available from the corresponding author upon reasonable request.
